# A Survey of Seasonal Gastrointestinal Parasitic Infections in Donkeys from a Semiarid Sub-Saharan Region, Sudan

**DOI:** 10.1155/2016/4602751

**Published:** 2016-05-19

**Authors:** Ahmed Abdurhman Ismail, Nasredin Khogali Ahmed, Ahmed Elhag Bashar, Hisham Ismail Seri, El Tigani Ahmed El Tigani-Asil, Adam Dawoud Abakar

**Affiliations:** ^1^Department of Pathology, Faculty of Veterinary Science, University of Nyala, P.O. Box 155, Nyala, Sudan; ^2^Directorate of Animal Health, State Ministry of Agriculture and Animal Resources, P.O. Box 155, Nyala, South Darfur, Sudan; ^3^Department of Microbiology and Parasitology, Faculty of Veterinary Science, University of Nyala, P.O. Box 155, Nyala, Sudan; ^4^College of Veterinary Medicine, Sudan University of Science and Technology, Khartoum, Sudan; ^5^Faculty of Agriculture and Veterinary Medicine, Qassim University, P.O. Box 6622, Buraidah 51452, Saudi Arabia; ^6^Department of Medical Parasitology, Faculty of Medical Laboratory Sciences, University of Gezira, P.O. Box 20, Wadmedani, Sudan

## Abstract

Out of 92 donkeys examined for gastrointestinal parasites, 90 animals were found infected by one or more gastrointestinal parasites with an overall prevalence rate of 97.78%. The distributions of the recovered parasites in the different parts of the body were as follows: stomach, 92.4%, small intestine, 19.6%, caecum, 88%, colon, 80.4%, rectum, 73.9%, and cranial mesenteric artery, 64.1%. A significant difference was found between mean parasite counts and seasons. Hot wet season had higher mean parasites count (5411.5 ± 1694.4) in comparison with hot dry (1795.9 ± 399.6) and cool dry (1719.9 ± 522.4) seasons. Although there was no significant difference between age and mean parasite count, animals more than four years old had high mean count (3361.3 ± 921.8) in comparison with 2330 ± 744.3 and 2030.2 ± 873.1 for young and adults animals, respectively. No significant positive or negative correlation was found between total parasite counts of infected animals and any of the climatic factors. The parasites identified were* Habronema* spp. (40.2%),* Trichostrongylus axei* (30.4%),* Parascaris equorum* (18.5%),* Anoplocephala perfoliata* (4.35%),* Gastrodiscus aegyptiacus* (8.7%), large strongyles (84%), small strongyles (72%), and* Oxyuris equi* (1.1%).

## 1. Introduction

The importance of donkeys in the Sudan is unequivocal. The animal provides support and transport at a low cost for urban and rural areas. The donkey has a potential of making valuable contribution to new development strategies such as reconstruction and development programmes in many parts of Sudan especially Darfur region as they diversify source of income in the rural areas. Despite the economic importance of donkeys in the Sudan, little attention has been drawn towards their diseases in general and particularly gastrointestinal parasites. The earliest recognized parasitic disease of donkeys in the Sudan was strongyle infections which have been shown to cause serious losses in affected areas [1, 2]. The report of Hamid et al. [3] from South Darfur has attracted the attention of scientists towards the importance of donkey parasites especially gastrointestinal parasites. A few years later, reports from Khartoum and Nyala provided some information on the distribution of gastrointestinal parasites and other parasitic diseases in the Sudan [4–7]. Nevertheless, the current situation of parasitic disease in other states where the donkey is usually prevalent is rather obscure and deserves some more emphasis. The present study reports on the prevalence of gastrointestinal parasites of donkeys in Nyala, South Darfur State, and identifies the gastrointestinal parasites infesting donkeys in the study area. Furthermore, the study highlights risk factors associated with gastrointestinal parasitic infection.

## 2. Materials and Methods

### 2.1. Study Area

**Figure 1:**
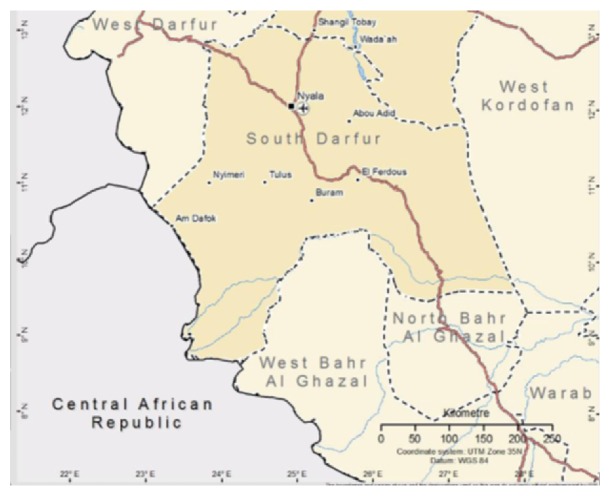
Geographical location of the study area, South Darfur State [8].

The present study was conducted at Nyala town, South Darfur State, Sudan. South Darfur State is located in the southwest of Sudan. It covers 139800 km^2^ between latitude 13–9.30° north and longitude 27–24.30° east. The state has common boarders with North Darfur, West Kordofan, Northern Bahr El Ghazal, and West Darfur States. It also shared boarders with Chad and Central Africa Republic [8]. The climate in South Darfur State is savannah type with clay sandy soil in the south, while the north is semidesert with sandy soil. The meteorological annual data of 40 years obtained from Nyala Airport Meteorological Station showed that the mean minimum and maximum temperature are 20.98° and 35.14°C. The mean annual relative humidity is 35.58% and the mean total rainfall is 402 mm. There is a single rainy season, which occurs between June and October, but the bulk of the rainfall takes place during the period of July–September. The area is traversed by several watercourses originating from Jebel Marra Mountains; the natural pasture is dominated by* abo-asabei* grass (*Dactyloctenium aegyptium*) with variable proportion of legumes (Figure 1).

### 2.2. Study Animals

**Figure 2:**
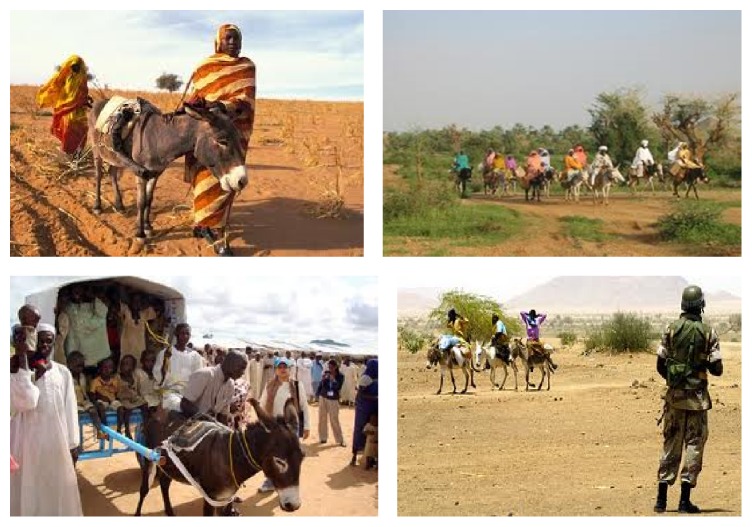
Climate and landscape of the study area. Pictures by the authors, 2011.

This study involved 92 donkeys. Animals used in this study were purchased from Nyala livestock market and were admitted from different areas in the South Darfur State but most of them came from camps of displaced people. All the animals examined were of common local type and of varying ages (1–13 years) and of both sexes (Figure 2). The majority were adult animals. Considering the husbandry and care practices of donkeys in South Darfur State, the body condition of the animals and their other parasitic faunas suggested that they had rarely received any drug treatment against gastrointestinal parasites.

### 2.3. Experimental Design

This is a cross-sectional study; the plan of work constituted a 12-month parasitological survey on the donkeys obtained from Nyala livestock market extending from May 2005 to April 2006. The examined animals were acquired at different seasons of the year representing the dry cold season (November–February), dry hot season (March–June), and wet hot season (July–October). They were from animals that graze on natural pasture throughout the year. Five to 13 donkeys were examined every month. The intervals between the acquisition of animals and necropsy examination range between 3 and 7 days.

### 2.4. Postmortem Procedures

From May 2005 to April 2006, 92 donkeys were killed and necropsied at the Faculty of Veterinary Science, University of Nyala. The animals were killed by the jugular vein and carotid artery bleeding after administration of either chloral hydrate or thiopentone sodium as general anesthesia. Every week 1-2 donkeys were sacrificed for postmortem examination for a whole year. The animals were fastened for two days prior to necropsy. The necropsy procedures were done as those previously described [9]. The collected materials from the different parts of the thoracic and abdominal cavities were sieved through 80 *μ*m mesh screen, the residues preserved in 10% formalin. The identification was accomplished by using binocular microscope under 4, 10, and 40x (Olympus microscope, Japan). The classification of nematodes was done using the early description. The identification of the cestodes and the trematodes was done as described by Soulsby [10].

### 2.5. Statistical Analysis

Data were summarized in terms of prevalence, abundance, and intensity of infection. Differences among prevalence rates in relation to season, age, and type were tested by Duncan test and the differences were considered significant when *P* < 0.05. Software used was SPSS for Windows, version 14.0.

## 3. Results

**Figure 3:**
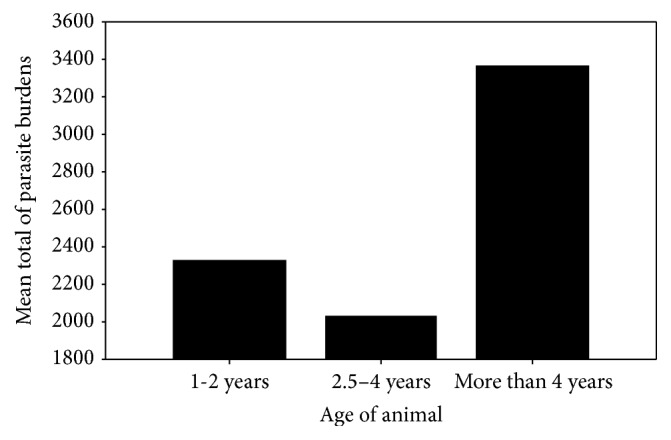
Effect of age on the parasitic burden on infected donkeys.

**Figure 4:**
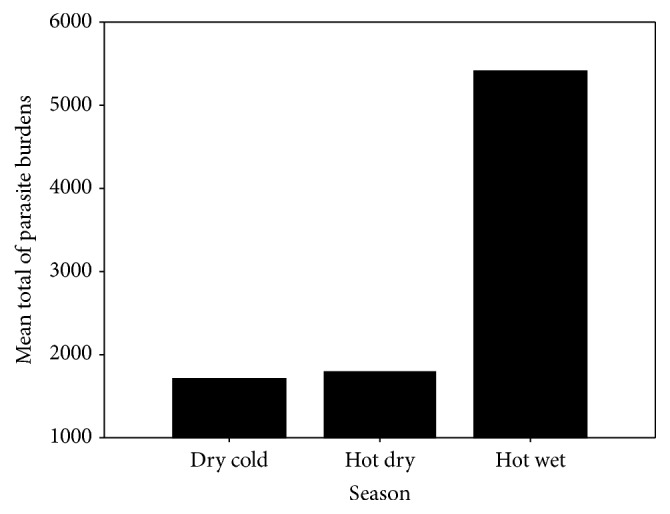
Seasonal prevalence of parasite burdens recorded from infected donkeys.

The postmortem examination of 92 donkeys for the presence of gastrointestinal parasites showed that 90 animals were infected with one or more parasites. The overall infection rate of identified parasites of donkeys obtained from Nyala livestock market was 97.78%. Almost all animals infected harboured mixed infections. The distributions of the recovered parasites in the different parts of the body were as follows: stomach (92.4%), small intestine (19.6%), caecum (88%), colon (80.4%), rectum (73.9%), and cranial mesenteric artery (64.1%). The effect of season on the prevalence rates is shown in Table 1. Although dry cold season gave 100% prevalence rate, no significant difference was observed between different seasons. The prevalence rate of identified parasites according to age is shown in Table 2. No significant difference was observed between the age groups and the prevalence of infection. Although there was no significant difference between age and mean parasite burdens, animals more than four years old had high mean burdens (3361.3 ± 921.8) in comparison with 2330 ± 744.3 and 2030.2 ± 873.1 for young and adult, respectively (Figure 3). Moreover, significant difference in mean parasite burdens was found among seasons (*r* = 0.263^*∗*^). Hot wet season had higher mean burdens (5411.5 ± 1694.9) in comparison with 1795.9 ± 399.6 and 1719.9 ± 522.4 for hot dry and dry cold seasons, respectively (Figure 4).

The mean total count of parasites recovered from small stomach, small intestine, colon, caecum, and rectum was depicted in Tables 3 [Table tab4]–5.

**Figure 5:**
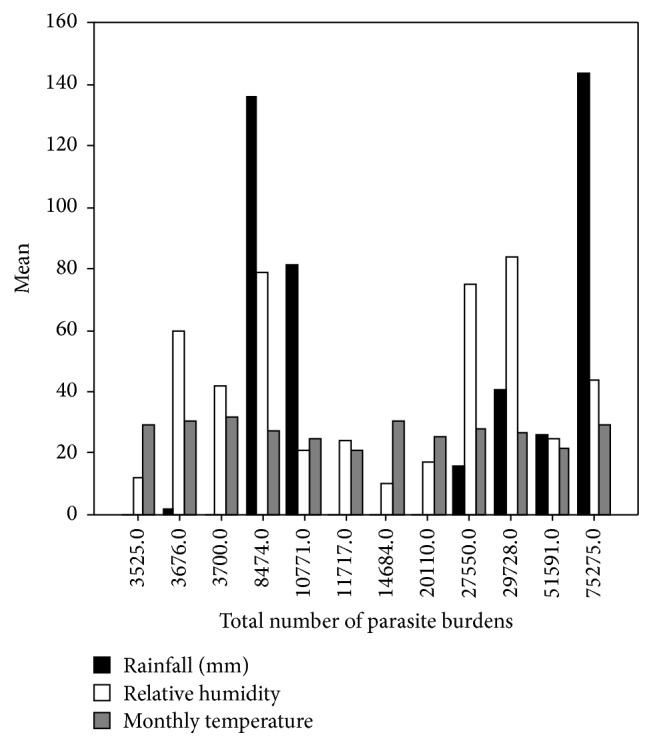
Effect of climatic factors on the internal parasitic burdens recovered from examined donkeys.

**Figure 6:**
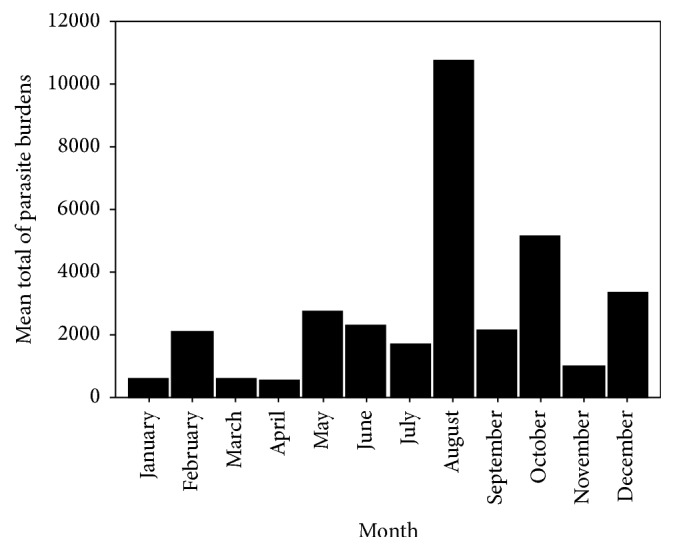
Monthly prevalence of parasite count among examined donkeys.

No significant positive or negative correlation was found between total parasitic count in infected animals and any of the climatic factors (Figure 5). The hot wet season is most preferable for development and survival of the recovered parasites. The highest mean total parasitic count was recorded in August while the least mean parasitic count was recorded in March and April (Figure 6). A total of 11 species and one genus of endoparasites were recovered from the donkeys in the current survey. They are* Habronema muscae* (56%),* Habronema microstoma* (37%), and* Habronema megastoma* (7%),* Trichostrongylus axei* (30.4%),* Parascaris equorum* (22.8%),* Anoplocephala perfoliata* (15.5),* Strongylus vulgaris* (60%),* Strongylus equinus* (22%), and* Strongylus edentatus* (18%), cyathostomins spp. (72.3%),* Gastrodiscus aegyptiacus* (4.5%), and* Oxyuris equi* (1.1%).

## 4. Discussion

The Darfur region is one of the most heavily populated regions with animals especially equines. However, there are no systemic surveys or records of parasitic diseases occurrences and prevalence. Nonetheless, there have been a few studies that were undertaken in the Darfur region. Previous studies on the evidence of the occurrence of equine gastrointestinal parasites have been provided by S. M. Kheir and H. S. M. Kheir [1], Eisa et al. [11], Hamid et al. [3], and Mohammed [5]. However, their data were based only on analysis of veterinary records, short-period abattoirs surveys, or fecal egg count from clinical cases brought for treatment at the veterinary educational hospitals. Donkey and other domestic animals are known to play a major role in disease transmission in Sudan in general and the study area in particular [6, 7, 12]. It is worth mentioning that human domesticated animals interphase reactions also contributed to maintain some bacterial infections [13] and viral infections [11] worldwide. The current study supported the previous observations on equine gastrointestinal infections in this area and further determined the magnitude of worm burden in apparently healthy donkeys by providing further information on their seasonal prevalence and the influence of the external environment on both the worm species composition and abundance on the host over 12 consecutive months in Nyala, South Darfur State.

The present study established that donkeys in South Darfur State harboured a high rate of gastrointestinal parasites throughout the year; only two animals were found free from internal parasites. This may be attributed to exposure of those animals to previous administration of anthelmintics drugs; 90 animals were positive. Mixed infections were detected in (97.7%) of the donkeys examined. This is a very high rate compared to the work of Ayele et al. [14], or S. M. Kheir and H. S. M. Kheir [1]; this may be attributed to the different methods that have been adopted; necropsy always gives a complete epidemiological picture. The high infection rate of parasitic helminthes affecting donkeys reported here might be associated with lack of anthelmintics intervention or might be due to mixed grazing and overcrowding which facilitate contamination between the different animals.

The overall prevalence of* Habronema* spp. was 40.21%. August recorded a higher mean number of worm burdens and (100%) prevalence. This could be attributed to adequate moisture and appropriate temperature that are favourable for both survival and propagation of the parasite and vectors as well. The identification of this parasite to species level indicated that* Habronema muscae* is dominant over other species. Similar result has been reported [15]. The intensity of infection with spirurids in the present work was similar to those reported in other equids from South Africa [16] and North Africa.

The prevalence of* Trichostrongylus axei* reported in this study was 64%; this finding is in agreement with the work of Feseha and Aweke [17] which reported 100% prevalence in donkeys of Ethiopia and Pandey et al. [18] who reported 93.5% in Morocco. The prevalence of* Trichostrongylus axei* reported here was very high when compared to the work of Seri et al. [4] in Sudanese donkey or Lyons et al. [19] in USA. This could be attributed to different methods of diagnosis and species of animal involved. These authors utilized fecal examination, while the current research depended solely on postmortem technique.

The prevalence of* Parascaris equorum* reported from this survey was 19.6%, which is in accordance with results stated by Yoseph et al. [20] and Fikru et al. [21] who gave 15.7% and 17.3% prevalence rates, respectively. Infection of donkeys with* Parascaris equorum* reported here is relatively high when compared to the work of Seri et al. [4] which reported that the prevalence rate of* Parascaris equorum* was 10.7% in the donkeys of Khartoum State and S. M. Kheir and H. S. M. Kheir [1] who found 6.6% in Nyala and 6.8% at Bahr El Arab by coprological examination, while, in Kenya, Mukhwana obtained 20.7% almost similar results [22]. The result of the current survey was very low when compared to the work of Feseha et al. [23] which reported 33% in Ethiopia and Garber [24] who found that 72% of examined donkeys in Chad were infested with* Parascaris equorum*; similarly, Abdelkarim [25] stated that the prevalence rate of the parasites among donkeys of Morocco is 37%.

The level of* Parascaris equorum* infection during the rainy season had a significant difference compared to the other seasons; this could be because of the adverse conditions prevailing during the hot season. Soulsby [10] stated that hotness dry conditions and direct sunlight kill the eggs in few weeks. Clayton [26] showed that a minimum of 80–83 days are required for newly laid eggs of* Parascaris equorum* to reach maturity and appear in small intestine so mature* Parascaris* is unexpected to be seen in the intestine at least at the beginning of the rainy season.

Prevalence rate of* Anoplocephala perfoliata* (3.3%) recorded in the present study is relatively low compared to the results of Tolliver et al. [27] which reported 17% from 513 horses in Kentucky. In some geographical areas like USA and Brazil, a necropsy examination showed a prevalence of infection as high as 60% [28, 29]. This low prevalence could be due to seasonality of oribatid mites vector [10].

The prevalence of small strongyles (cyathostomins) reported here was in accordance with findings of Abdelkarim [25] and Feseha et al. [23]. This result was relatively higher than the one reported by Mukhwana [22]. These nematodes are the most common helminthes parasites of equines and can cause considerable morbidity and mortality [30]. Infections with these species of cyathostomins have been recorded in horses, donkeys, and zebra worldwide [31, 32]. Research activities on these nematodes are high because larval cyathostominosis is a serious syndrome that leads to fatal severe colitis. Moreover, resistance to anthelmintics within cyathostomins has been reported widely. Therefore, adoption of accurate diagnostic methods for identification of small strongyles in the study area is highly recommended.

Strongyles infection was correlated with very high prevalence rate, 88%, in comparison to previous reports [4, 33, 34]. The present study confirmed that strongyle infestation was significantly higher in the rainy season of the year. This result was in accordance with the work of Yoseph et al. [20], Mulate [35], and Fikru et al. [21] which indicated that fecal egg count began to rise to severe levels during the wet period of the year. From this seasonal variation of strongyle infestation, a treatment schedule could be proposed. A treatment can be given at the end of the rainy season. At this time, the animals are well nourished and may harbour a large number of parasites without being seriously affected. Eliminating of these parasites will improve the adaptation of the animals to the harsh dry season conditions. Another treatment can be prescribed at the end of the dry season. This treatment reduces infestation of pasture at the first rainfall by residual parasites.

The lower prevalence rate (4.3%) of* Gastrodiscus aegyptiacus* reported in the current study might be due to adverse ecological conditions for development of the intermediate host snail,* Bulinus forskalii*, which need permanent humidity, water marshes, dams, and dampness [10]. The three positive cases found in this study mostly originated from migratory nomads that spend their summer season at Bahr El Arab where the ecological conditions are favourable for the intermediate host of* Gastrodiscus aegyptiacus*.* Gastrodiscus aegyptiacus* is more prevalent in the colon than the caecum (1 : 15); accordingly, it could be said that the parasite favours colon of the donkey compared to the caecum.

The prevalence of* Oxyuris equi* reported here is very low when compared to the findings of Tolliver et al. [27] and Yoseph et al. [20]. The low prevalence rate in this study might be due to the effect of relative high temperature in the study area that desiccates the highly susceptible* Oxyuris equi* eggs.

In conclusion, donkeys in South Darfur, most of them coming from camps of displaced people, are highly affected by a wide range of gastrointestinal parasites that are prevailing throughout the year. It seems that environmental conditions are favourable for both larval and adult stages, which lead to parasitic burden.

## Figures and Tables

**Table 1:** Effect of season on the prevalence of donkey parasite recovered by postmortem examination during May 2005–April 2006.

Seasons	Number of animals examined	Positive numbers	Prevalence %
Dry cold	27	27	100
Hot dry	37	36	97.3
Hot wet	28	27	96.4

Overall means	92	90	97.8

**Table 2:** Prevalence of recovered donkey parasites according to animal age.

Ages	Number of animals examined	Positive numbers	Prevalence %
1-2 years	23	22	95.7
2.5–4 years	17	17	100
>4 years	52	51	98.1

Overall	92	90	97.8

**Table 3:** Monthly mean count ± SEM of parasites recovered from stomach and small intestine of necropsied donkeys.

Months	*Habronema* spp.	*Parascaris equorum*	*Trichostrongylus axei*	*Anoplocephala perfoliata*
January	0	5 ± 2	0	0
February	0	3.7 ± 1.2	34	0
March	0	2	2	0
April	0	2	47	0
May	186.7 ± 86.7	1.5 ± 0.5	0	0
June	34 ± 9.3	4.6 ± 1.9	40.3 ± 20.7	0
July	80	0	10 ± 4.7	1
August	108.9 ± 33	0	38.5 ± 15.8	0
September	28.6 ± 9.8	0	43.5 ± 8.8	0
October	69.3 ± 13.9	14	11.5 ± 2.6	1
November	43.7 ± 29.4	2	3.7 ± 1.8	2
December	41.7 ± 24.2	17.5 ± 15.5	18 ± 11	2

Overall	70.1 ± 12.2	5.7 ± 1.8	24.3 ± 4.5	1.5 ± 19.6

**Table 4 tab4:** Monthly mean worm count ± SEM of parasites recovered from caecum of necropsied donkeys.

Months	Large strongyle species	*Cyathostomum* spp.	*Gastrodiscus aegyptiacus*
January	143.2 ± 59.6	20.3 ± 15.4	0
February	265.3 ± 62.2	104 ± 41.7	0
March	177.6 ± 73.3	152.5 ± 77.5	0
April	152.5 ± 82.4	24 ± 16	0
May	194.9 ± 70.2	367.7 ± 155.6	0
June	373.2 ± 83.5	1012.7 ± 611	0
July	272.6 ± 159.5	57 ± 10	1470
August	705.3 ± 160.7	368.6 ± 91.1	0
September	314.4 ± 167	72.7 ± 70.1	45 ± 33
October	314.8 ± 60.1	38.8 ± 27.9	5
November	126.4 ± 41.9	37.8 ± 12.8	0
December	1272.2 ± 936.5	468.3 ± 173.6	0

Overall	357.6 ± 76.1	293.7 ± 85.1	391.3 ± 360

**Table 5:** Monthly mean worm count ± SEM of parasites recovered from colon of necropsied donkeys.

Months	Large strongyle species	*Cyathostomum* spp.	*Gastrodiscus aegyptiacus*
January	144.3 ± 33.6	241.3 ± 101.8	0
February	463.4 ± 147.3	1366.7 ± 574.7	0
March	160 ± 73.8	173.5 ± 112.9	0
April	170.8 ± 63.8	335 ± 118.6	0
May	410.2 ± 221.6	2357.6 ± 1012.7	9
June	1073 ± 934.6	1153.9 ± 504.7	2460
July	272.7 ± 189	620 ± 580	2350
August	4106.6 ± 2579.2	5768.5 ± 3138.7	0
September	139.6 ± 49.6	1606.3 ± 1383	207.7 ± 147.5
October	2412.9 ± 2084.9	2350.8 ± 1094.1	5 ± 2
November	163.6 ± 29.6	1270.8 ± 651.2	0
December	1272.2 ± 936.5	468.3 ± 173.6	0

Overall	1038.4 ± 392.5	1739.1 ± 394.5	681.3 ± 380.8
